# LIFE SPACE MOBILITY AND NEIGHBORHOODS: HOW HOME MODIFICATIONS IMPACT AGING IN PLACE

**DOI:** 10.1093/geroni/igz038.935

**Published:** 2019-11-08

**Authors:** Alan DeLaTorre, Ivis García, Julianne Reno, Ja Young Kim, Keith Diaz Moore

**Affiliations:** 1 Portland State University, Portland, Oregon, United States; 2 University of Utah, Salt Lake City, Utah, United States

## Abstract

This presentation details a mixed methods study funded by the National Institute for Transportation and Communities that was conducted with 50 older adults aged 65 and older who lived in Portland, Oregon (n=25) and Salt Lake County, Utah (n=25). The purpose of the study was to improve understanding of how home modifications affect older adults’ mobility in different life-spaces (e.g., one’s bedroom, neighborhood), their understanding of aging in place and neighborhood, and their ability to age in place. During each home visit, a series of research protocols (i.e., surveys, interviews, mapping exercises) were carried out with each participant. The study found that home modifications (e.g., grab bars, replacing showers with bathtubs, and adding raised toilets) were reported to increase in-home mobility and, for some, their independence; however, for certain participants, those same modifications were less useful, especially to those with the need for caregiver supports. Life-space mobility outside the home was impacted by home modification such as ramps and railings on stairs; for some, those modifications bolstered social connections and access to services. Overall, home modification were seen as enabling both mobility and aging in place. Furthermore, respondents’ understanding and description of their neighborhoods varied greatly and were influenced by mobility barriers (e.g., presence of sidewalks and crosswalks) and available amenities.

